# Supplementary material for: Differences in regional brain structure in toddlers with autism are related to future language outcomes
Source: Nat Commun. 2024 Jun 13;15:5075. doi: 10.1038/s41467-024-48952-4 (PMC11176156; doi:10.1038/s41467-024-48952-4)
Supplement: Supplementary file 1 — Supplementary Information [file 41467_2024_48952_MOESM1_ESM.pdf]

Differences in regional brain structure in toddlers with autism are related to future language outcomes

Kuaikuai Duan[1*], Lisa Eyler[2,3], Karen Pierce[1], Michael V. Lombardo[4], Michael Datko[1], Donald J. Hagler, Jr.[5], Vani Taluja[1], Javad Zahiri[1], Kathleen Campbell[1], Cynthia Carter Barnes[1], Steven Arias[1], Srinivasa Nalabolu[1], Jaden Troxel[1], Peng Ji[6], Eric Courchesne[1*]

[1]Autism Center of Excellence, Department of Neurosciences, University of California, San Diego, La Jolla, CA, 92037, USA

[2]Department of Psychiatry, University of California, San Diego, La Jolla, CA, 92093, USA

[3]VISN 22 Mental Illness Research, Education, and Clinical Center, VA San Diego Healthcare System, San Diego, CA, 92161, USA

[4]Laboratory for Autism and Neurodevelopmental Disorders, Center for Neuroscience and Cognitive Systems @UniTn, Istituto Italiano di Tecnologia, Rovereto, 38068, Italy.

[5]Center for Multimodal Imaging and Genetics, Department of Radiology, University of California, San Diego, La Jolla, CA, 92093, USA

[6]Department of Chemistry and Biochemistry, University of California, San Diego, La Jolla, CA, 92093, USA

Supplementary Information

Sex-stratified ASD vs. TD brain structure differences in main sample

We tested ASD vs. TD differences in regional brain structure in males and females separately using linear mixed effect models while adjusting effects from age, brain global measurements (fixed effects) and longitudinal scans (random intercept and slope for each subject). The test results are listed in Table S1. The identified ASD vs. TD volume differences still show up in both males and females in cerebellum, posterior CC, and the four cortical subregions (LH fusiform, LH and RH middle temporal, and RH caudal anterior cingulate). Moreover, compared to TD, ASD toddlers have smaller surface area in RH caudal anterior cingulate and smaller cortical thickness in RH pars opercularis in both female and males. The remaining subregional surface area and cortical thickness differences were present in males but not females.

Table S1. ASD vs TD brain structure differences stratified by sex. Two-tailed linear mixed effect models were used, with adjusting effects from age, brain global measurements (fixed effects) and longitudinal scans (random intercept and slope for each subject). P values listed in the table were raw values and were not adjusted for multiple comparison.

ASD vs. TD difference	male (274 scans, 185 from ASD)		female (98 scans, 43 from ASD)	
	p	Cohen's d	p	Cohen's d
**Non-cortical volume**				
Right Cerebellum Cortex	0.04	-0.27	0.03	-0.45
Posterior CC	2.84E-2	0.31	2.54E-2	0.52
Mid Anterior CC	0.12	0.22	2.47E-2	0.55
Mid Posterior CC	4.68E-2	0.28	0.32	0.24
Anterior CC	0.29	0.15	3.15E-2	0.54
**Cortical volume**				
LH Fusiform	7.87E-3	0.36	6.14E-3	0.61
LH Middle Temporal	4.96E-2	0.26	5.99E-4	0.90
RH Caudal Anterior Cingulate	6.87E-3	-0.36	1.25E-2	-0.60
RH Middle Temporal	4.63E-3	0.39	5.06E-4	0.76
**Cortical surface area**				
RH Caudal Anterior Cingulate	1.73E-3	-0.42	2.06E-3	-0.75
RH Medial Orbitofrontal	9.54E-5	-0.53	0.11	-0.38
RH Posterior Cingulate	6.58E-4	-0.48	0.81	0.05
**Cortical thickness**				
LH Superior Temporal	2.35E-5	0.58	0.51	0.15
LH Pars Opercularis	9.90E-3	-0.35	0.08	-0.39
LH Caudal Middle Frontal	2.78E-3	-0.40	0.62	-0.12
LH Pericalcarine	0.22	-0.17	0.10	-0.38
RH Pars Opercularis	1.91E-3	-0.44	0.03	-0.48
RH Bank SSTS	2.98E-3	0.41	0.18	0.35

ASD vs. TD brain structure differences for initial MRI scans

We included only the initial scan for each of the 275 toddlers and then tested ASD vs TD difference while controlling the effects from age, sex and global brain measures (eTIV, total surface area or mean cortical thickness). Tables S2-S4 listed the corresponding results, we can observe that most

of the previously identified brain measures still show ASD vs. TD differences, although some are with weaker effect sizes compared to that from inclusion of repeated scans, but all except mid-posterior corpus callosum and anterior corpus callosum have *p* values less than 0.05.

Table S2, ASD vs. TD difference of initial regional volume. Two-tailed linear regression models were used, with controlling the effects from age, sex, and brain size (eTIV). P values listed in the table were raw values and were not adjusted for multiple comparison.

Regions	p values	Cohen's d
LH Fusiform	9.48E-03	0.35
LH Middle Temporal	4.80E-03	0.38
RH Caudal Anterior Cingulate	1.82E-02	-0.32
RH Middle Temporal	6.02E-04	0.46
Right Cerebellum Cortex	4.50E-03	-0.38
Posterior CC	1.55E-02	0.33
Mid Posterior CC	0.21	0.17
Mid Anterior CC	4.17E-02	0.27
Anterior CC	0.09	0.22

Table S3, ASD vs. TD difference of initial regional surface area. Two-tailed linear regression models were used, with controlling the effects from age, sex, and total surface area. P values listed in the table were raw values and were not adjusted for multiple comparison.

Regions	p values	Cohen's d
RH Caudal Anterior Cingulate	2.37E-03	-0.41
RH Medial Orbitofrontal	4.48E-04	-0.48
RH Posterior Cingulate	7.37E-03	-0.36

Table S4, ASD vs. TD difference of initial regional cortical thickness. Two-tailed linear regression models were used, with controlling the effects from age, sex, and mean thickness. P values listed in the table were raw values and were not adjusted for multiple comparison.

Regions	p values	Cohen's d
LH Pars Opercularis	3.31E-02	-0.29
LH Superior Temporal	5.20E-04	0.47
RH Bank SSTS	4.09E-02	0.28
RH Pars Opercularis	1.68E-02	-0.32

Age square effect on ASD vs. TD brain structure differences in main sample

To account for possible nonlinear age effects, we included age^2 as a covariance in addition to age, sex, and global brain measures (eTIV, total surface area or mean cortical thickness). Tables S5-S7 show the corresponding p and cohen’s d values for identified regional volume, surface area and thickness measurements. We can see that most of the previously identified brain measures still show significant ASD vs. TD differences, and all have p values less than 0.05.

Table S5, ASD vs. TD difference of regional volume with controlling age square effect. Two-tailed linear mixed effect models were used, with adjusting effects from sex, age, age^2, eTIV (fixed effects) and longitudinal scans (random intercept and slope for each subject). P values listed in the table were raw values and were not adjusted for multiple comparison.

Regions	p values	Cohen's d
LH Fusiform	2.20E-04	0.42
LH Middle Temporal	1.79E-03	0.36
RH Caudal Anterior Cingulate	9.21E-04	-0.38
RH Middle Temporal	6.79E-05	0.46
Right Cerebellum Cortex	8.11E-03	-0.31
Posterior CC	1.62E-03	0.37
Mid Posterior CC	2.02E-02	0.27
Mid Anterior CC	9.91E-03	0.30
Anterior CC	3.16E-02	0.25

Table S6, ASD vs. TD difference of regional surface area with controlling age square effect. Two-tailed linear mixed effect models were used, with adjusting effects from sex, age, age^2, total surface area (fixed effects) and longitudinal scans (random intercept and slope for each subject). P values listed in the table were raw values and were not adjusted for multiple comparison.

Regions	p values	Cohen's d
RH Caudal Anterior Cingulate	5.18E-05	-0.47
RH Medial Orbitofrontal	2.77E-05	-0.49
RH Posterior Cingulate	9.20E-04	-0.39

Table S7, ASD vs. TD difference of regional cortical thickness with controlling age square effect. Two-tailed linear mixed effect models were used, with adjusting effects from sex, age, age^2, mean thickness (fixed effects) and longitudinal scans (random intercept and slope for each subject). P values listed in the table were raw values and were not adjusted for multiple comparison.

Regions	p values	Cohen's d
LH Pars Opercularis	1.60E-03	-0.36
LH Superior Temporal	3.21E-04	0.42
RH Bank SSTS	1.27E-03	0.38
RH Pars Opercularis	2.61E-04	-0.43

Language outcome differences between ASD Low/Average and TD

Since our ASD Low/Average toddlers were significantly older than TD toddlers (p = 5.15E-8) and female/male ratio was also not balanced between ASD Low/Average (10/59) and TD (44/65) toddlers (p = 2.54E-4), we tested language outcome differences between ASD Low/Average and TD toddlers using an N-way ANOVA test while controlling age, sex, and interaction effects between grouping label (ASD Low/Average or TD) and age and sex (i.e., grouping × age, grouping × sex). We did not observe significant ASD Low/Average vs. TD differences for both Mullen ELT (p = 0.61) and RLT (p = 0.35) at outcome visit.

Associations between brain structures and behavior in TD toddlers

Although knowing whether typically developing toddlers also have brain growth variation that indexes variations of autism symptom severity and cognitive deficits, is clearly not of clinical interest or utility, we tested this in the interests of fuller understanding of possible brain-behavior patterns in TD at very early ages. Thus, significant brain-behavior associations identified in ASD toddlers were also examined in TD toddlers using the same model as for ASD toddlers. Interaction between brain and diagnosis was further examined by adding brain×diagnosis and diagnosis terms in the regression model. Correction of multiple comparisons was not applied for TD toddlers. Associations with $p < 0.05$ were reported below:

Brain-behavior association directions in TD toddlers were opposite to those seen in ASD (Fig. S6). *Larger* GMV in LH middle temporal was significantly associated with *higher* Mullen ratio VR scores (r (95% CI) = 0.23 (0.03, 0.41), $p = 0.02$, Fig. S7). Larger GMV in mid anterior CC was significantly related to higher Mullen ratio RL scores (r (95% CI) = 0.22 (0.02, 0.40), $p = 0.03$, Fig. S8).

GMV in LH fusiform and LH middle temporal (MT) regions significantly interacted with diagnosis to predict Mullen ELC (fusiform: $p = 0.01$; MT: $p = 2.73 \times 10^{-3}$), Mullen ratio RL (fusiform: $p = 3.74 \times 10^{-3}$; MT: $p = 1.18 \times 10^{-2}$), and Mullen ratio VR (fusiform: $p = 2.33 \times 10^{-3}$; MT: $p = 2.79 \times 10^{-4}$). GMV in posterior CC ($p = 0.03$) and mid anterior CC ($p = 0.04$) significantly interacted with diagnosis to predict Mullen ratio EL. Associations were strongly negative in the ASD group, but near zero or positive in the TD group. SA in RH caudal anterior cingulate significantly interacted with diagnosis to predict Mullen ratio RL ($p = 1.52 \times 10^{-2}$). Scatter plots of significant brain-behavior associations are presented in Fig. S6.

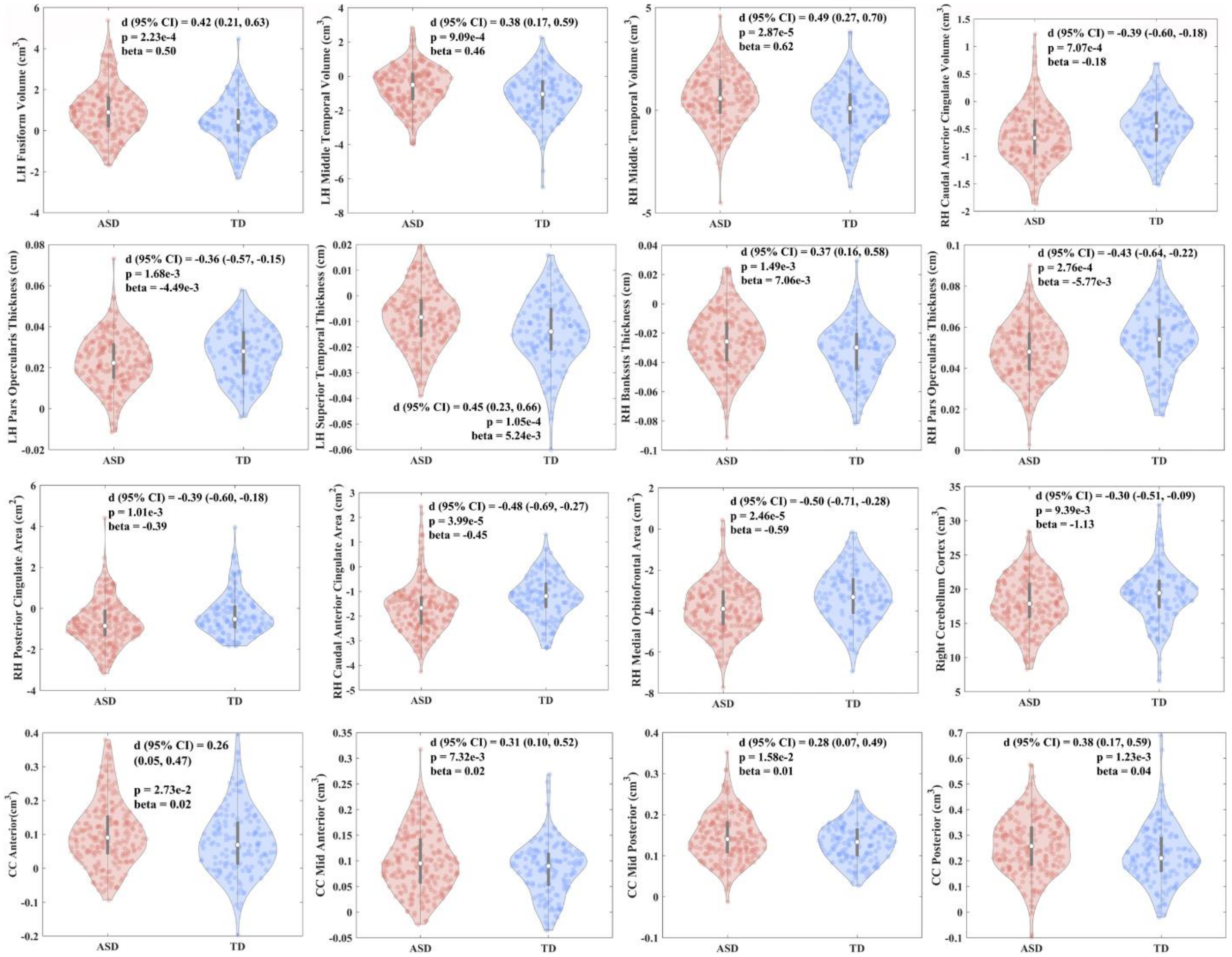

Figure S1 Violin plots of brain regions showing significant difference between ASD and TD toddlers in *discovery* samples. P, Cohen's d and its 95% confidence interval (95% CI), and beta values of ASD vs TD difference are displayed for each brain region. ASD and TD toddlers were presented as medium light shades of red and cyan-blue, respectively (the same for Figures S2, S4, and S5). Note that the brain measure of a specific region displayed on y axis was adjusted for the fixed effect from age and sex and the random effect from longitudinal scans (the same for Figure S2). Given that we coded diagnosis as a dummy variable (ASD = 1, TD = 0), the beta value for diagnosis can be interpret as how much larger/smaller (unit: cm for thickness, cm^2 for SA, cm^3 for volume) ASD toddlers' brain is compared to TDs' in a specific region (the same for Figure S2).

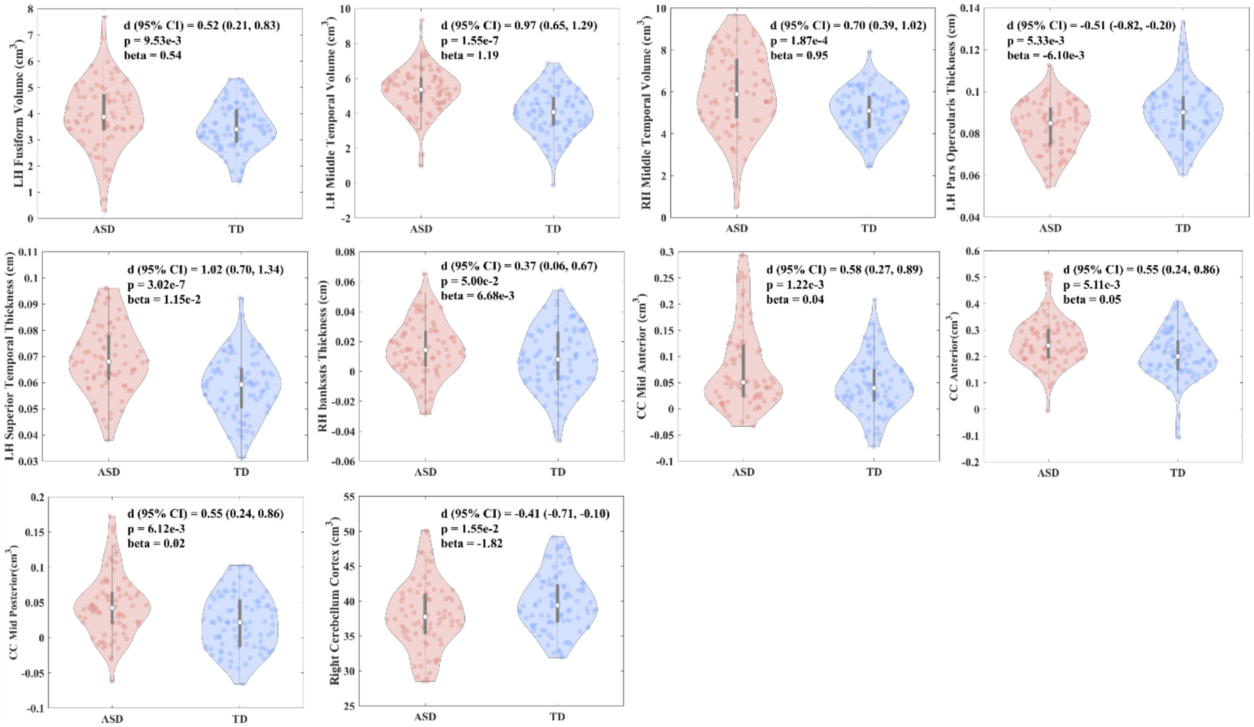

Figure S2 Violin plots of brain regions that were replicated for ASD vs TD differences in *replication* samples. P, Cohen's d and its 95% confidence interval (95% CI), and beta values of ASD vs TD difference are also presented for each brain region.

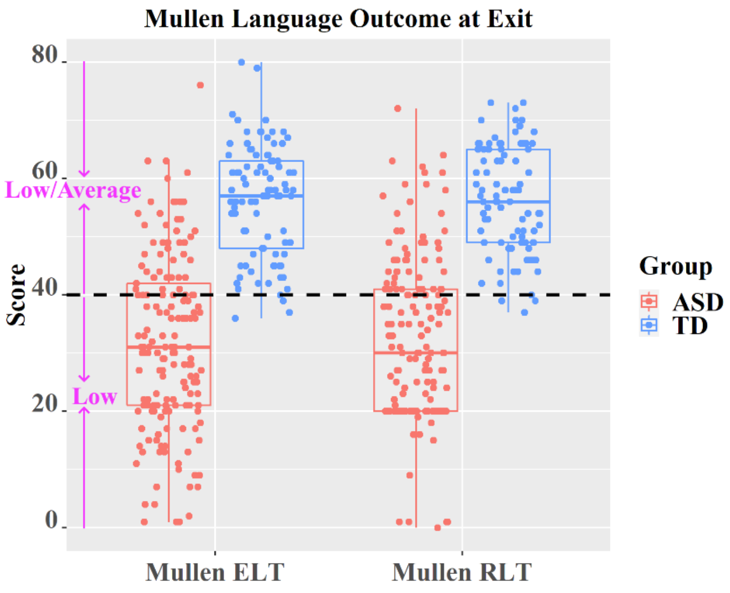

Figure S3 Box plot of Mullen ELT and RLT scores of TD and ASD toddlers with Low/Average or Low language outcome. The dash line indicates a score of 40 for Mullen ELT and RLT.

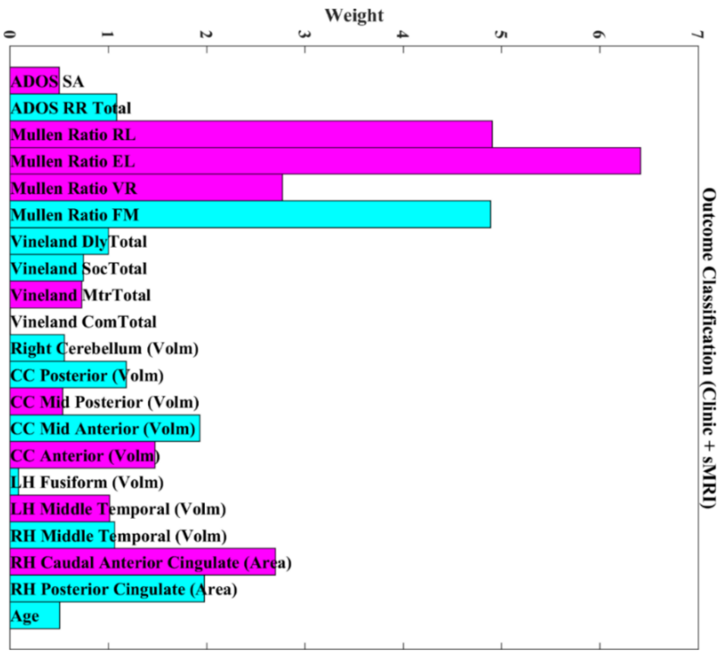

Figure S4 Weights of intake clinic and sMRI features for predicting language outcome of ASD toddlers. Larger values of intake feature with magenta color associated with better language outcome, while larger values of intake features with cyan color related to poorer language outcome.

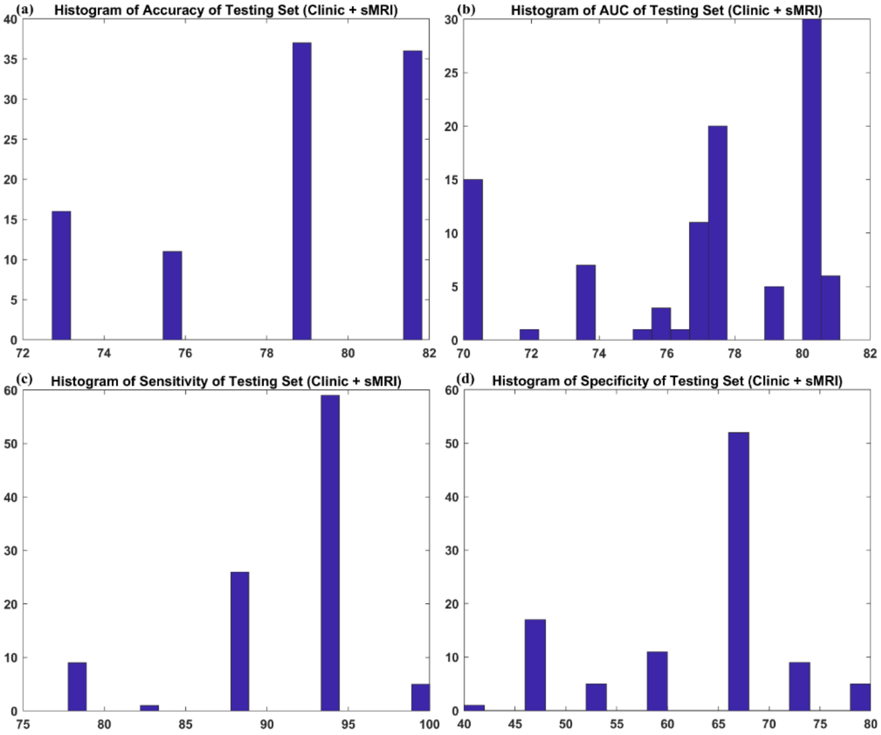

Figure S5 Histogram of (a) accuracy, (b) AUC, (c) sensitivity, and (d) specificity values from 100 iterations of 5-fold cross-validation for the Clinic + sMRI model.

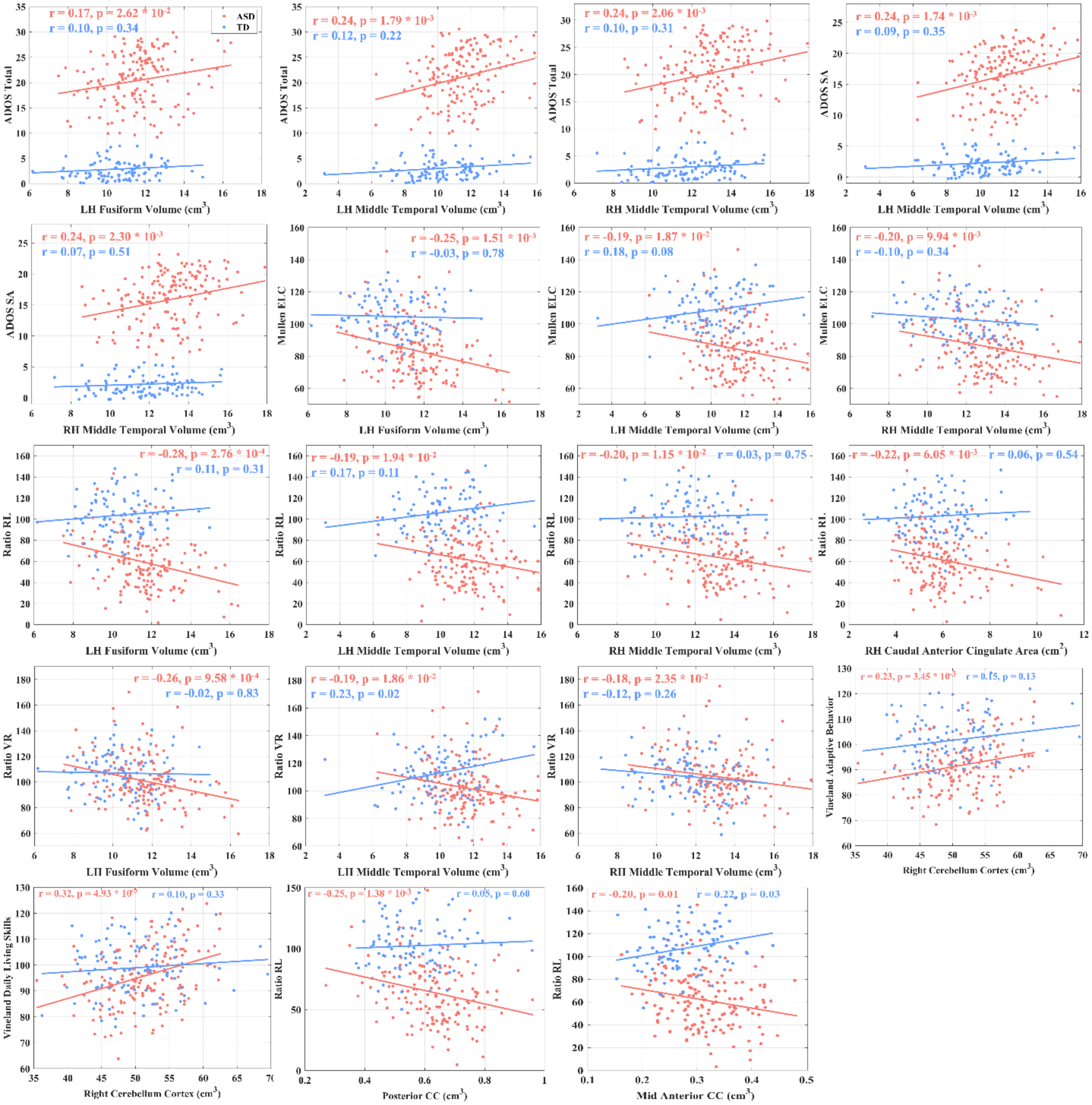

Figure S6 Scatter plots for significant brain-behavior associations. X axis represents a brain measure (volume/SA in a specific brain region), and y axis represents a behavioral measure (e.g., ADOS total, ADOS SA, Mullen ELC, Mullen ratio RL, Mullen ratio VR, Vineland adaptive behavior, and Vineland daily living skills). Note that the behavioral measure displayed on y axis was adjusted for age and sex effects. Dots with red color indicate ASD toddlers and dots with blue color indicate TD toddlers.

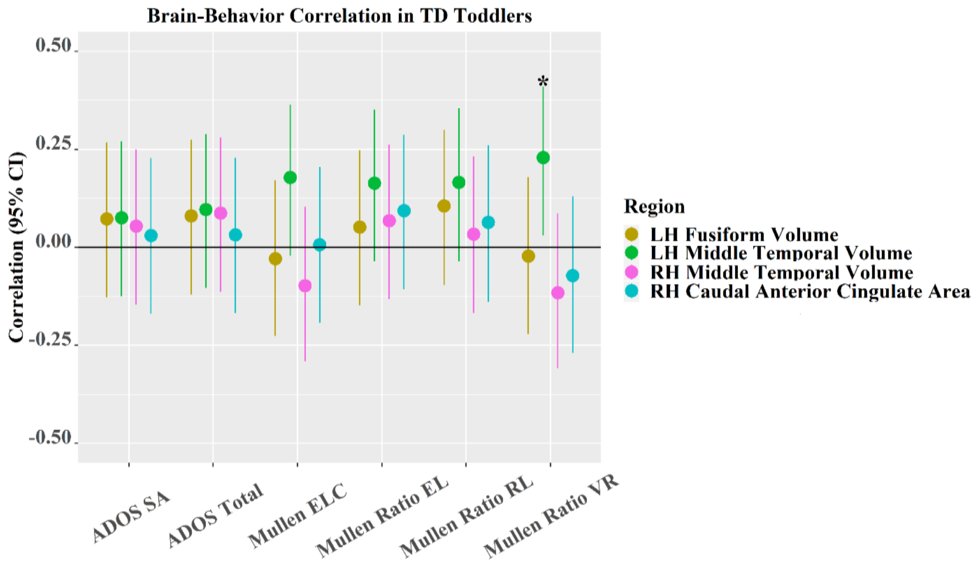

Figure S7 Association between behavior and ASD discriminating cortical brain regions and its 95% CI in TD toddlers. Note that * indicates the correlation is significant; colors of medium dark shades of yellow, green, cyan and a medium light shade of magenta denote LH fusiform volume, LH middle temporal volume, RH caudal anterior cingulate SA, and RH middle temporal volume, respectively. N=106 independent TD toddlers were tested for associations with ADOS subscales. 98-101 independent TD toddlers were examined for associations with Mullen subscales. Each dot represents the true correlation value, and the error bar represents its 95% confidence interval.

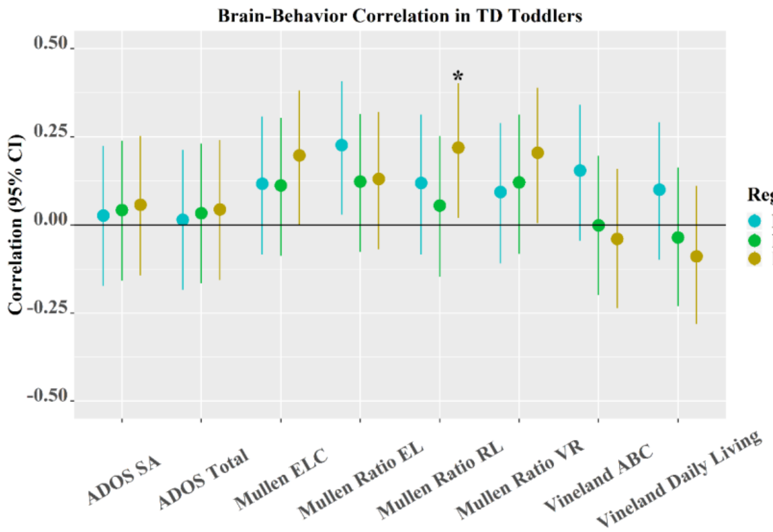

Figure S8 Association between behavior and ASD discriminating non-cortical brain regions in TD toddlers and its 95% CI. Note that * indicates the correlation is significant; colors of cyan, green, and medium dark shades of yellow denote right cerebellum, posterior CC and mid anterior CC, respectively. N=106 independent TD toddlers were tested for associations with ADOS subscales. N=107 independent TD toddlers were tested for associations with Vineland subscales. 98-101 independent TD toddlers were examined for associations with Mullen subscales. Each dot represents the true correlation value, and the error bar represents its 95% confidence interval.

Table S8, Statistical results of ASD Low vs. TD and ASD Low/Average vs. TD differences for brain regions showing significant GMV differences between ASD and TD. Two-tailed linear mixed effect models were used, with adjusting effects from sex, age, eTIV (fixed effects) and longitudinal scans (random intercept and slope for each subject). P values listed in the table were raw values and were not adjusted for multiple comparison.

GMV Features	ASD Low vs. TD		ASD Low/Average vs. TD	
	p	Cohen's d	p	Cohen's d
Right Cerebellum Cortex	3.88×10^{-2}	-0.27	1.90×10^{-2}	-0.34
Posterior CC	1.42×10^{-4}	0.51	0.63	0.07
Mid Posterior CC	1.53×10^{-3}	0.43	0.66	0.07
Mid Anterior CC	4.34×10^{-4}	0.45	0.99	1.46×10^{-3}
Anterior CC	1.13×10^{-2}	0.33	0.90	0.02
LH Fusiform	1.11×10^{-5}	0.58	0.13	0.21
LH Middle Temporal	6.10×10^{-4}	0.45	0.05	0.28
RH Caudal Anterior Cingulate	8.37×10^{-3}	-0.35	1.60×10^{-3}	-0.45
RH Middle Temporal	8.50×10^{-8}	0.74	0.51	0.09

Table S9, Statistical results of ASD Low vs. TD and ASD Low/Average vs TD differences for brain regions showing significant SA differences between ASD and TD. Two-tailed linear mixed effect models were used, with adjusting effects from sex, age, total surface area (fixed effects) and longitudinal scans (random intercept and slope for each subject). P values listed in the table were raw values and were not adjusted for multiple comparison.

SA Features	ASD Low vs. TD		ASD Low/Average vs. TD	
	p	Cohen's d	p	Cohen's d
RH Caudal Anterior Cingulate	7.09×10^{-4}	-0.46	2.35×10^{-4}	-0.52
RH Medial Orbitofrontal	2.12×10^{-6}	-0.64	3.22×10^{-2}	-0.30
RH Posterior Cingulate	7.60×10^{-3}	-0.36	2.43×10^{-4}	-0.56

Table S10, Statistical results of ASD Low vs. TD and ASD Low/Average vs TD differences for brain regions showing significant thickness differences between ASD and TD. Two-tailed linear mixed effect models were used, with adjusting effects from sex, age, mean thickness (fixed effects) and longitudinal scans (random intercept and slope for each subject). P values listed in the table were raw values and were not adjusted for multiple comparison.

Thickness Features	ASD Low vs. TD		ASD Low/Average vs. TD	
	p	Cohen's d	p	Cohen's d
LH Pars Opercularis	6.57×10^{-3}	-0.36	1.09×10^{-2}	-0.37
LH Superior Temporal	1.01×10^{-3}	0.43	5.97×10^{-4}	0.49
RH Bank SSTS	3.37×10^{-3}	0.40	3.68×10^{-3}	0.43
RH Pars Opercularis	1.44×10^{-4}	-0.52	0.06	-0.27

Supplementary Methods

Sex-stratified clinical test scores in main sample

Table S11 summarizes the clinical test scores stratified by sex. Given that sex collinears with diagnosis, and males are relatively older than female toddlers in main sample, we tested male vs. female differences for ADOS/Mullen/Vineland subscale scores using an N-way ANOVA tests

including diagnosis and age as covariates. We found that ADOS/Vineland and most Mullen (except Mullen ELC) subscale scores did not show significant female vs. male differences. Only Mullen ELC showed a male vs. female difference, where females have higher ELC scores than males (p = 0.04).

Table S11. Intake clinical test scores for male and female toddlers in main sample.

Characteristics	male (202 toddlers)	female (73 toddlers)	p value (male vs. female)
**Demographics at MRI and clinical visit**			
Number of ASD/TD	137/65	29/44	2.60×10^{-5} [a]
Age at clinical visit (years)	2.25 (0.76)	2.07 (0.75)	0.09 [b]
Age at MRI scan (years)	2.36 (0.76)	2.23 (0.71)	0.20 [b]
**ADOS (module T, 1 or 2) score**	**male (N =202)**	**female (N = 72)**	
ADOS SA	9.87 (6.74)	7.17 (6.85)	0.24 [c]
ADOS RRB	2.73 (2.30)	1.74 (2.31)	0.99 [c]
ADOS Total	12.60 (8.60)	8.90 (8.87)	0.34 [c]
**Mullen score**	**male (N = 192-194)**	**female (N = 68-70)**	
Ratio fine motor (ratio FM) [d]	93.18 (20.50), N = 193	104.05 (18.33), N = 69	0.13 [c]
Ratio visual reception (ratio VR) [d]	94.60 (22.17), N = 192	108.24 (23.35), N = 69	0.07 [c]
Ratio expressive language (ratio EL) [d]	75.08 (26.62), N = 193	91.92 (31.50), N = 70	0.14 [c]
Ratio receptive language (ratio RL) [d]	77.51 (29.91), N = 193	94.92 (34.17), N = 68	0.28 [c]
Early learning composite (ELC)	83.65 (23.80), N = 194	100.57 (26.57), N = 70	0.04 [c]
**Vineland standard score**	**male (N = 202)**	**female (N = 73)**	
Adaptive behaviour composite	87.37 (13.90)	94.77 (16.09)	0.52 [c]
Daily living skills	89.64 (13.70)	97.55 (14.67)	0.09 [c]
Socialization	88.49 (14.83)	94.96 (15.84)	0.72 [c]
Motor skills	93.71 (11.17)	96.10 (12.01)	0.68 [c]
Communication	85.39 (17.23)	93.29 (18.48)	0.61 [c]

[a]Pearson's chi-squared test.

[b]Welch's two sample t test.

[c]N-way ANOVA test including age and sex as covariates.

[d]Mullen subscale ratio score was computed by dividing the age equivalent score of that subscale by the toddler's chronological age.

Note, all statistical tests were two-tailed. Values for age and all clinical test scores are presented as mean (SD). SD represents standard deviation. ADOS SA represents ADOS social affect, and ADOS RRB presents ADOS restricted and repetitive behavior. ADOS, Mullen and Vineland are evaluated at the same clinic visit. Mullen subscale ratio score was computed by dividing the age equivalent score of that subscale by the toddler's chronological age.

Distribution and time points of MRI scans in main sample

The included 275 toddlers were initially scanned at a mean age of 27.98 months and longitudinally followed up at mean ages of 37.05, 44.04 and 55.10 months, respectively. The distribution of MRI scans was summarized in Table S12.

Table S12, The distribution of MRI scans in the main sample.

	1 scan	2 scans	3 scans	4 scans
Subject #	187	80	7	1

Quality rating of MRI scan and segmentation

Each scan was visually checked by two independent raters. Scans with obvious motion strips (see Fig. S9 for an example) were flagged.

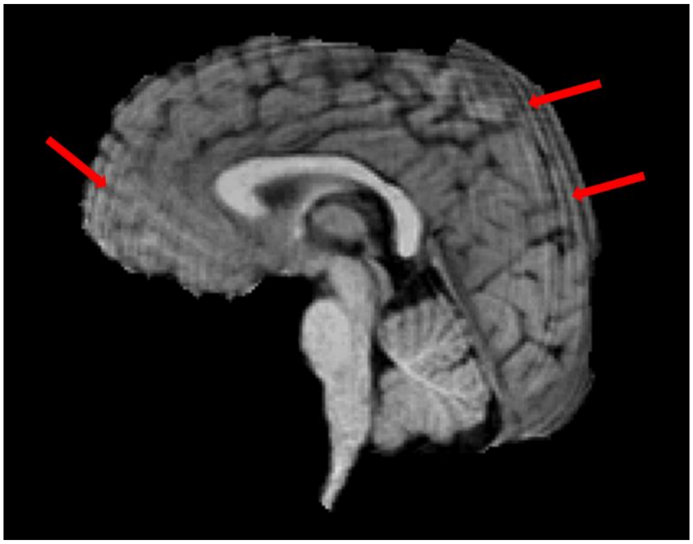

Figure S9 An example scan that was excluded due to excessive motion stripes. Arrows point to motion stripes.

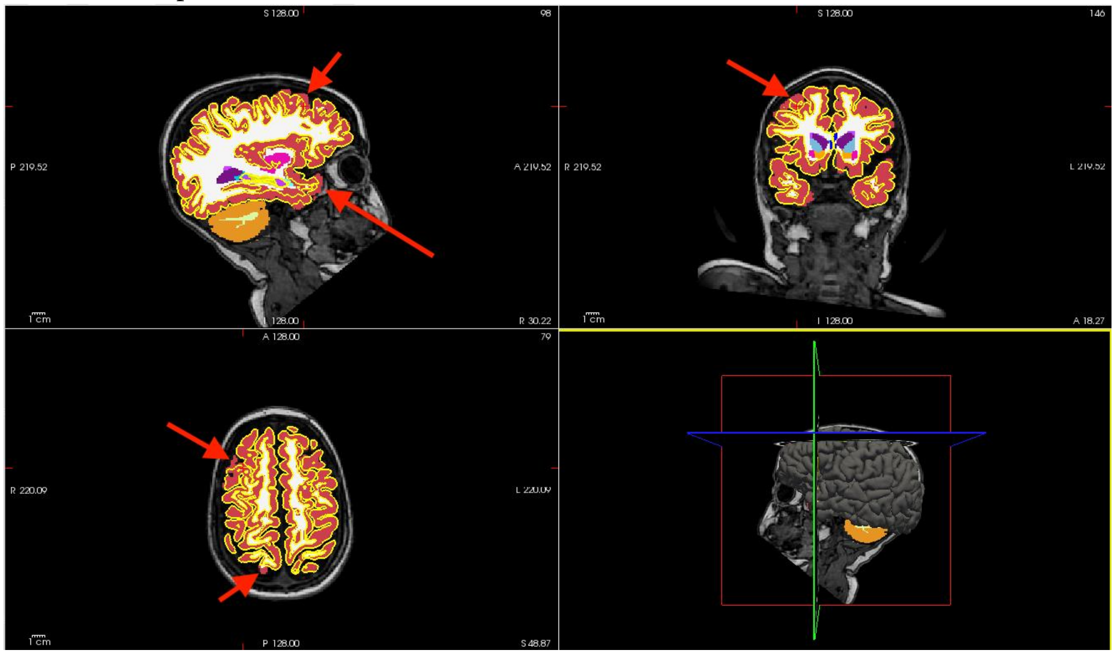

Figure S10 An example scan that was excluded due to bad segmentation quality. Arrows demonstrate regions with poor matches between surfaces and aseg.

Among scans with poor sMRI or bad segmentation quality, 49.23% were from ASD toddlers and 50.77% from TD toddlers, indicating no group difference between ASD and TD. The inter-rater reliability for quality rating is estimated as 0.64 (CI: [0.48, 0.75]) using two-way random effects ANOVA model [1, 2]. Scans with highly visible motion stripes in T1w image or partial coverage of

the brain were marked as poor quality. Bad segmentation quality refers to poor match between surfaces and aseg in multiple large regions of cortex (see Fig. S10 for an example).

Supplementary References

1. Shrout PE, Fleiss JL. Intraclass correlations: uses in assessing rater reliability. *Psychological bulletin* **86**, 420 (1979).

2. Koo TK, Li MY. A Guideline of Selecting and Reporting Intraclass Correlation Coefficients for Reliability Research. *J Chiropr Med* **15**, 155-163 (2016).
