# Supplementary material for: Differences in regional brain structure in toddlers with autism are related to future language outcomes
Source: Nat Commun. 2024 Jun 13;15:5075. doi: 10.1038/s41467-024-48952-4 (PMC11176156; doi:10.1038/s41467-024-48952-4)
Supplement: Supplementary file 2 — Reporting Summary [file 41467_2024_48952_MOESM2_ESM.pdf]

nature portfolio

Corresponding author(s): Kuaikuai Duan, Eric Courchesne

Last updated by author(s): Mar 25, 2024

Reporting Summary

Nature Portfolio wishes to improve the reproducibility of the work that we publish. This form provides structure for consistency and transparency in reporting. For further information on Nature Portfolio policies, see our Editorial Policies and the Editorial Policy Checklist.

Statistics

For all statistical analyses, confirm that the following items are present in the figure legend, table legend, main text, or Methods section.

n/a	Confirmed	
☐	☒	The exact sample size (*n*) for each experimental group/condition, given as a discrete number and unit of measurement
☐	☒	A statement on whether measurements were taken from distinct samples or whether the same sample was measured repeatedly
☐	☒	The statistical test(s) used AND whether they are one- or two-sided<br>*Only common tests should be described solely by name; describe more complex techniques in the Methods section.*
☐	☒	A description of all covariates tested
☐	☒	A description of any assumptions or corrections, such as tests of normality and adjustment for multiple comparisons
☐	☒	A full description of the statistical parameters including central tendency (e.g. means) or other basic estimates (e.g. regression coefficient) AND variation (e.g. standard deviation) or associated estimates of uncertainty (e.g. confidence intervals)
☐	☒	For null hypothesis testing, the test statistic (e.g. *F*, *t*, *r*) with confidence intervals, effect sizes, degrees of freedom and *P* value noted<br>*Give P values as exact values whenever suitable.*
☒	☐	For Bayesian analysis, information on the choice of priors and Markov chain Monte Carlo settings
☒	☐	For hierarchical and complex designs, identification of the appropriate level for tests and full reporting of outcomes
☐	☒	Estimates of effect sizes (e.g. Cohen's *d*, Pearson's *r*), indicating how they were calculated

Our web collection on statistics for biologists contains articles on many of the points above.

Software and code

Policy information about availability of computer code

Data collection	No software was used for data collection.
Data analysis	Open-source Freesurfer 5.3 (https://surfer.nmr.mgh.harvard.edu/) was used to segment all structural MRI images. Existing functions in Matlab 2020b were used to perform analyses included in this paper. Specifically, 'fitlmematrix.m' was used to fit linear mixed-effect model on each brain region to compute ASD vs. TD difference; 'regstats.m' was applied to assess brain-behavior associations; 'fitclinear.m' and 'kfoldLoss.m' were used to train the language outcome classification model and 'predict.m' was used to predict language outcome for the independent testing samples. R 4.1.2 was used to plot Fig. 1-3. Publicly available R package 'ggseg' was used to plot Figs.1-2.

For manuscripts utilizing custom algorithms or software that are central to the research but not yet described in published literature, software must be made available to editors and reviewers. We strongly encourage code deposition in a community repository (e.g. GitHub). See the Nature Portfolio guidelines for submitting code & software for further information.

Data

Policy information about availability of data

All manuscripts must include a data availability statement. This statement should provide the following information, where applicable:

- Accession codes, unique identifiers, or web links for publicly available datasets
- A description of any restrictions on data availability
- For clinical datasets or third party data, please ensure that the statement adheres to our policy

Raw sMRI and clinical data are available from the National Institute of Mental Health Data Archive (collection ID = 9). The processed data are available from Dr.

Courchesne upon reasonable request.

Human research participants

Policy information about studies involving human research participants and Sex and Gender in Research.

Reporting on sex and gender	Samples were collected from both males and females. The sex of sampled individuals was determined by parental report. Since we our main goal is to characterize brain difference at diagnostic group (ASD or TD) level rather than the individual level, we used all available scans from females and males in the main analyses. Additionally, we performed sex-stratified ASD vs. TD brain difference tests to examine whether the identified regional difference also present in males and females, separately.
Population characteristics	In the main sample, clinical and behavioral scores and sMRI scans were collected from 275 toddlers (166 ASD, 109 TD; 202 male, 73 female; mean age = 2.35 years). Diagnosis of ASD is determined by highly experienced and licensed psychologists using diagnostic criteria in DSM 5 in combination with the gold-standard ADOS evaluation. TD toddlers showed no history of any developmental delay. Out of 275 toddlers, 187 had only an intake sMRI scan collected, 88 had one or more follow-up sMRI scans collected at intervals ranging from 0.5 to 27 (mean ± standard deviation: 13.03 ± 3.35) months after the initial/ previous scan, contributing to 372 scans in total. Out of 166 ASD toddlers, 157 had a Mullen evaluation at the outcome visit and were stratified into two outcome groups: ASD Low/Average (N = 69; 59 males, 10 females; age = 33.88 ± 4.44 months) and ASD Low (N = 88; 71 males, 17 females; age = 34.55 ± 5.18 months). These 157 ASD toddlers were used for language outcome prediction analysis.<br><br>In the replication sample, 75 unique toddlers (38 ASD, 37 TD; 55 male, 20 female) were included, contributing to 167 test-retest scans.
Recruitment	Toddlers were recruited through community referral and a population-based screening method in collaboration with pediatricians via the Get SET Early Approach, formally known as the 1-Year Well-Baby Check-Up Approach. Toddlers who received their initial diagnostic and clinical evaluations at < 36 months were invited to return for repeat evaluations until they reached 48 months. We are not aware of any self-selection or other biases that are likely to impact the recruitment of our cohort.
Ethics oversight	This study was approved by the University of California, San Diego Institutional Review Board.

Note that full information on the approval of the study protocol must also be provided in the manuscript.

Field-specific reporting

Please select the one below that is the best fit for your research. If you are not sure, read the appropriate sections before making your selection.

[x] Life sciences [ ] Behavioural & social sciences [ ] Ecological, evolutionary & environmental sciences

For a reference copy of the document with all sections, see nature.com/documents/nr-reporting-summary-flat.pdf

Life sciences study design

All studies must disclose on these points even when the disclosure is negative.

Sample size	Sample sizes were not pre-determined by statistical methods; our cohort of 275 toddlers (166 ASD, 109 TD) is a robust sample size based on the replication results and previous MRI studies (Courchesne et al., 2007, Neuron, 56, 399–413; Schumann et al., 2010, Journal of Neuroscience, 30, 4419–4427; Carper et al., 2005, Biological psychiatry, 57, 126-133; Lombardo et al., 2021, Science advances, 7, eabh1663); we also include a replication data set collected from 75 toddlers (38 ASD and 37 TD) to examine the replicability of identified regional brain differences. Plus, a total of 539 test-retest MRI scans (372 from main samples and 167 from replication samples) were included in the study, which is the largest MRI scan sample among all previous MRI studies of ASD toddlers to date.
Data exclusions	In the main sample, 68 out of 343 subjects were excluded due to poor MRI scan quality or scans with bad segmentation quality. In replication sample, one out of 76 toddlers was excluded due to poor MRI scan quality or scans with bad segmentation quality.
Replication	Leveraging test-retest MRI scans from an independent replication dataset of 75 toddlers (38 ASD and 38 TD), we replicated the majority of the identified brain volume and thickness differences. Group difference of the identified three cortical surface area measures were not replicated.
Randomization	Random allocation of participants to groups is not applicable because diagnostic labels (i.e., ASD and TD) have been identified prior to the study. No other randomization procedures were implemented as part of the data collection process.
Blinding	Data collection and analyses were not performed blind to the conditions of the experiment.

Reporting for specific materials, systems and methods

We require information from authors about some types of materials, experimental systems and methods used in many studies. Here, indicate whether each material, system or method listed is relevant to your study. If you are not sure if a list item applies to your research, read the appropriate section before selecting a response.

Materials & experimental systems

n/a	Involved in the study
☒	☐ Antibodies
☒	☐ Eukaryotic cell lines
☒	☐ Palaeontology and archaeology
☒	☐ Animals and other organisms
☒	☐ Clinical data
☒	☐ Dual use research of concern

Methods

n/a	Involved in the study
☒	☐ ChIP-seq
☒	☐ Flow cytometry
☐	☒ MRI-based neuroimaging

Magnetic resonance imaging

Experimental design

Design type	Structural MRI (T1); no task was performed.
Design specifications	One structural MRI scan was collected during one session.
Behavioral performance measures	Behavioral performance measures are not collected during the scan.

Acquisition

Imaging type(s)	Sturctural
Field strength	1.5 T
Sequence & imaging parameters	Structural MRI data were collected with a T1-weighted IR-FSPGR (inversion recovery fast-spoiled prepared gradient recalled) sagittal protocol with TE (echo time) = 2.8 ms, TR (repetition time) = 6.5 ms, flip angle = 12°, bandwidth = 31.25 kHz, field of view = 24 cm, and slice thickness = 1.2 mm.
Area of acquisition	Whole brain was scanned.

Diffusion MRI ☐ Used ☒ Not used

Preprocessing

Preprocessing software	Freesurfer 5.3
Normalization	Normalization was not performed prior to segmentation since we are interested in the raw regional brain volume.
Normalization template	No normalization template was used.
Noise and artifact removal	Noise and motion artifact were visually evaluated by two independent raters with a rating scale ranging from 0 to 3 (0=best, 1=great, 2=usable, 3=unusable). Scans with unusable rating were removed from this study.
Volume censoring	Volume censoring was not applied to this study.

Statistical modeling & inference

Model type and settings	Multivariate regression and machine learning models were employed to analyze the data. Linear mixed effect models were used to compute regional brain mophormetry differences between ASD and TD.
Effect(s) tested	Alterations of regional brain volume/cortical surface area/cortical thickness in ASD compared to TD were identified using linear mixed effect models. Multivariate regression analyses were used to evaluate brain-behavior associations. Support vector machine with ridge regularization was applied to predict later language outcome.

Specify type of analysis: ☐ Whole brain ☒ ROI-based ☐ Both

Anatomical location(s): Anatomical locations were decided by Freesurfer 5.3.

Statistic type for inference (See Eklund et al. 2016)	Cluster/regional-wise measurements were used, including regional brain volume, regional cortical surface area and cortical thickness.
Correction	FDR was applied to correct for multiple comparisons.

Models & analysis

n/a	Involved in the study
☒	☐ Functional and/or effective connectivity
☒	☐ Graph analysis
☐	☒ Multivariate modeling or predictive analysis

Multivariate modeling and predictive analysis

No dimension reduction was applied. Features of interest were extracted from freesurfer.
In the group difference analyses, each regional brain measurement was used as the dependent variable. Age, sex, brain global measurement and diagnosis were used as independent variables, where subject ID was modeled as random effect to account for test-retest scans (each subject has a random intercept and a random slope), and other independent variables were modeled as fixed effects.

Multivariate regression analyses were used for evaluating brain-behavior associations. The behavioral measures were treated as the dependent variable, age, sex, and regional brain measurement were modeled as independent variables.

Support vector machine with ridge regularization was used for predicting language outcome 6 months later. Each model was trained and cross-validated with the training samples (n = 124, 80% ASD samples) using 5-fold cross-validation, and its performance was evaluated with a untouched hold-out testing set (n = 33, 20% ASD samples). Accuracy, sensitivity, specificity, and area under the receiver operating characteristic (ROC) curve (AUC) were computed on the untouch hold-out testing set to reflect the performances of prediction models. 100 iterations of 5-fold cross-validation was performed to reflect the dispersion of the classification performance.
