# Supplementary material for: Differences in regional brain structure in toddlers with autism are related to future language outcomes
Source: Nat Commun. 2024 Jun 13;15:5075. doi: 10.1038/s41467-024-48952-4 (PMC11176156; doi:10.1038/s41467-024-48952-4)
Supplement: Supplementary file 4 — Description of Additional Supplementary Files [file 41467_2024_48952_MOESM4_ESM.pdf]

Title: Supplementary Data 1

Description: Brain regions showing nominal significant ASD vs. TD differences ($p < 0.05$) in the main sample. In this Table, brain regions (colume A), p values (column B) and corresponding Cohen's d values (column C) for ASD vs. TD difference are reported.
